# Supplementary material for: Altered Cytokine Response of Human Brain Endothelial Cells after Stimulation with Malaria Patient Plasma
Source: Cells. 2021 Jul 1;10(7):1656. doi: 10.3390/cells10071656 (PMC8303479; doi:10.3390/cells10071656)
Supplement: Supplementary file 1 [file cells-10-01656-s001.zip › Table S1.pdf]

Table S1 List of plasmas examined, indicating the donor's parasitemia

Malaria patient (M) / Healthy control (H)	Parasitemia (%)
M1	2
M2	1.5
M3	8
M4	3
M5	2
**M6***	3
M7	5
M8	1.5
**M9 ***	2.5
**M10***	4
**M11***	2.5
M12	4.5
M13	7
M14	8
M15	11
M16	<1%
M17	<1%
M18	2%
M19	6%
M20	<1%
M21	1%
M22	<1%
M23	1%
M24	1%
M25	3%
M26	1.5
M27	<1
H1	-
H2	-
H3	-
H4	-
**H5***	-
H6	-
H7	-
**H8***	-
H9	-
**H10***	-
H11	-
H12	-
H13	-
H14	-
H15	-
H16	-
H17	-
H18	-
H19	-
H20	-
H21	-
H22	-

* Investigation of the influence of plasma on the activation of HBEC-5i cells using a transcriptome approach. M6, M9, M10, M11: 4 biological replicates each; H5, H10: 2 biological replicates each; H8: 1 biological replicate.
