# Supplementary material for: Altered Cytokine Response of Human Brain Endothelial Cells after Stimulation with Malaria Patient Plasma
Source: Cells. 2021 Jul 1;10(7):1656. doi: 10.3390/cells10071656 (PMC8303479; doi:10.3390/cells10071656)
Supplement: Supplementary file 1 [file cells-10-01656-s001.zip › Table S2.pdf]

Table S2 Number of plasmas analysed from malaria patients and healthy individuals and number of culture supernatants analysed from HBEC-5i cells stimulated with individual plasma samples from malaria patients and healthy individuals.

Cytokine/Chemokine/ Growth Factor	Plasma		HBEC-5i Culture Supernatant	
	Healthy Controls (N)	Malaria Patients (N)	Healthy Controls (N)	Malaria Patients (N)
IL-1α	14	26	17	22
IL-1β	21	24	21	26
IL-6	21	20	21	26
IL-12p70	20	24	21	26
IFN-β	21	24	21	26
IL-1RA	14	26	17	22
IL-10	21	20	21	26
IL-11	13	26	17	22
CCL3/MIP1α	14	26	17	22
CCL20/MIP3α	13	26	17	22
CXCL1/Groα	14	26	10	6
CXCL5/ENA78	14	26	17	22
IL-8/CXCL8	21	20	15	16
CXCL10/IP-10	21	20	21	26
VEGF	14	26	17	22
