# Supplementary material for: Altered Cytokine Response of Human Brain Endothelial Cells after Stimulation with Malaria Patient Plasma
Source: Cells. 2021 Jul 1;10(7):1656. doi: 10.3390/cells10071656 (PMC8303479; doi:10.3390/cells10071656)
Supplement: Supplementary file 1 [file cells-10-01656-s001.zip › Table S7.pdf]

Table S7 Levels of cytokines in the plasma of three control individuals (H5, H8, H10) and of four malaria patients (M6, M9, M10, M11), which were used to stimulate endothelial cells (HBEC-5i) for transcriptome analysis.

**Cytokine/Chemokine Growth Factor**	**H5^{Plasma}**	**H8^{Plasma}**	**H10^{Plasma}**	**M6^{Plasma}**	**M9^{Plasma}**	**M10^{Plasma}**	**M11^{Plasma}**
	**pg/mL**						
IL-6	1.7	0	0	na	9.4	10.2	3.9
IL-1RA	0	0	0	38.6	244.8	2523.9	270
IL-10	19.3	0	0.7	na	260.3	234.6	399.8
IL-11	0	0	0	122.4	0	47.6	0
CCL3	21.4	31.2	42.1	107.1	45.4	126.8	22.9
CCL20	3.3	18.6	7	19.4	3.7	53.8	13.8
CXCL1	0	0	43.8	491.7	0	65.2	27.8
CXCL5	841.9	263.7	290.9	122.4	169.6	954.1	549
CXCL8/IL-8	8.2	0	1.7	na	19.7	3.8	1
CXCL10	481.6	13.2	5.4	na	774.4	468.4	228.8
VEGF	0	0	0	49.4	61	90.7	0

*M6: Malaria patient with a parasitemia of 3%
M9: Malaria patient with a parasitemia of 2.5%
M10: Malaria patient with a parasitemia of 4%
M11: Malaria patient with a parasitemia of 2.5%
na: values not available
