# Supplementary material for: Altered Cytokine Response of Human Brain Endothelial Cells after Stimulation with Malaria Patient Plasma
Source: Cells. 2021 Jul 1;10(7):1656. doi: 10.3390/cells10071656 (PMC8303479; doi:10.3390/cells10071656)
Supplement: Supplementary file 1 [file cells-10-01656-s001.zip › Table S8.pdf]

Table S8. Levels of cytokines in the supernatant of endothelial cells (HBEC-5i), stimulated with plasma from three control individuals (H5, H8, H10) and from four malaria patients (M6, M9, M10, M11), which were also used for transcriptome analysis of the plasma-stimulated HBEC-5i cells.

**Cytokine/Chemokine Growth Factor**	**H5^{SN}** pg/mL	**H8^{SN}**	**H10^{SN}**	**M6^{SN*}**	**M9^{SN}**	**M10^{SN}**	**M11^{SN}**
IL-6	200.3	994	581.8	excl.	1602.8	1264.2	1949.8
IL-RA	118.3	38.3	70.8	excl.	13.2	61.8	36.8
IL-11	422.7	134.9	139	excl.	437.2	547.3	564.6
CCL3	113.1	31.9	32.4	excl.	221.1	190.9	187.7
CCL20	5,17	0	0,9	excl.	13.7	19	23.8
CXCL1	5560.8	293.2	545.8	excl.	nd	nd	nd
CXCL5	105.3	31.9	53	excl.	142.5	209.6	270.8
CXCL8	762.5	6018	3626	excl.	12862	10180	13190
CXCL10	0	12.5	0	excl.	46.6	39.3	82.1
VEGF	120.9	31.6	52	excl.	182	167.4	194

*M6: Malaria patient with a parasitemia of 3%
M9: Malaria patient with a parasitemia of 2.5%
M10: Malaria patient with a parasitemia of 4%
M11: Malaria patient with a parasitemia of 2.5%
^{SN}Supernatant
nd: not detectable, values above detectable range of 10000 pg/mL
excl.: values excluded due to measurement error
